# Indomethacin and Diclofenac Hybrids with Oleanolic Acid Oximes Modulate Key Signaling Pathways in Pancreatic Cancer Cells

**DOI:** 10.3390/ijms23031230

**Published:** 2022-01-22

**Authors:** Maria Narożna, Violetta Krajka-Kuźniak, Robert Kleszcz, Wanda Baer-Dubowska

**Affiliations:** 1 Department of Pharmaceutical Biochemistry, Poznan University of Medical Sciences, 4, Święcicki Street, 60-781 Poznań, Poland; maria.narozna@ump.edu.pl (M.N.); vkrajka@ump.edu.pl (V.K.-K.); kleszcz@ump.edu.pl (R.K.); 2 Program in Cell Cycle and Cancer Biology, Oklahoma Medical Research Foundation, 825, NE 13th Street, Oklahoma City, OK 73104, USA

**Keywords:** diclofenac, indomethacin, oleanolic acid derivative conjugates, NF-κB, Nrf2, MAPKs, PSN-1 cells, inflammation, reactive oxygen species

## Abstract

Our earlier studies showed that coupling nonsteroidal anti-inflammatory drugs (NSAIDs) with oleanolic acid derivatives increased their anti-inflammatory activity in human hepatoma cells. The aim of this study was to evaluate their effect on the signaling pathways involved in inflammation processes in human pancreatic cancer (PC) cells. Cultured PSN-1 cells were exposed for 24 h (30 µM) to OA oxime (OAO) derivatives substituted with benzyl or morpholide groups and their conjugates with indomethacin (IND) or diclofenac (DCL). The activation of NF-κB and Nrf2 was assessed by the evaluation of the translocation of their active forms into the nucleus and their binding to specific DNA sequences via the ELISA assay. The expression of NF-κB and Nrf2 target genes was evaluated by R-T PCR and Western blot analysis. The conjugation of IND or DCL with OAO derivatives increased cytotoxicity and their effect on the tested signaling pathways. The most effective compound was the DCL hybrid with OAO morpholide (4d). This compound significantly reduced the activation and expression of NF-κB and enhanced the activation and expression of Nrf2. Increased expression of Nrf2 target genes led to reduced ROS production. Moreover, MAPKs and the related pathways were also affected. Therefore, conjugate 4d deserves more comprehensive studies as a potential PC therapeutic agent.

## 1. Introduction

Pancreatic cancer (PC) is a major cause of cancer-associated mortality worldwide, with a dismal overall prognosis that has remained practically unchanged for many decades. Prevention or early diagnosis at a curable stage is still exceedingly difficult; patients rarely exhibit symptoms, and tumors do not display sensitive and specific markers to aid detection [1]. 

Chronic inflammation is considered a significant risk factor of many cancers, and pancreatic cancer is one of them. During this process, upregulation of pro-inflammatory molecules and pathways provides a favorable microenvironment for the exponential growth of malignant cells. Therefore, inflammation may provide both the critical mutations resulting from the DNA damage, e.g., by reactive oxygen species (ROS), and create the proper environment to foster tumor growth [2].

The NF-κB and Nrf2 signaling pathways play key roles in regulating the body’s responses to stress and inflammation. Moreover, several lines of evidence suggest that these pathways play a crucial role in PC development, progression and resistance, and both transcription factors are often overexpressed in pancreatic cancer cells [3,4].

NF-κB comprises a family of conserved and structurally related proteins, including RelA/p65, Rel/cRel, RelB, NF-κB1/p50, and NF-B2/p52. When inactivated, NF-κB is found in the cytosol bound to an IκB inhibitor protein (IκBα). Through the involvement of membrane receptors, a variety of extracellular signals can activate the IκB kinase (IKK) enzyme. IKK, in turn, phosphorylates the IκBα protein, leading to its ubiquitination and degradation by the proteasome [5]. 

Nrf2 is associated with the Keap1 protein and resides in cytosol. In normal conditions, Keap1 targets Nrf2 for ubiquitylation, leading to its proteasomal degradation [6]. In response to chemical or oxidative stress, the interaction between Nrf2 and Keap1 is perturbed, resulting in the stabilization and nuclear accumulation of Nrf2 [7,8,9]. The Nrf2 in the nucleus interacts with antioxidant response elements (ARE) in the promoter regions of a plethora of genes coding for phase 2 detoxifying enzymes (e.g., glutathione-S-transferases and NAD(P)H quinone oxidoreductase), antioxidant enzymes (e.g., SOD and GPx) and several other cytoprotective proteins [10,11,12,13]. 

For a long time, Nrf2 was considered an attractive and effective target for chemopreventive strategies [14,15] to maintain electrophilic and cellular redox balance. Through the induction of antioxidative target genes, Nrf2 could protect non-transformed cells against DNA damage and, thereby, may prevent mutagenesis. However, there is growing evidence that Nrf2 activation can lead to tumor development and enhanced chemoresistance once a malignant transformation occurs. Stable overexpression of Nrf2 results in the enhanced resistance of cancer cells to chemotherapeutics [16].

The interplay between these pathways occurs through a range of complex molecular interactions and can often depend on the cell type and tissue context. These interactions operate via both transcriptional and posttranscriptional mechanisms, allowing dynamic responses to ever-changing environmental cues. Despite convincing evidence for functional interactions between the Nrf2 and NF-κB pathways, many aspects of the conditional and dynamic nature of crosstalk remain unknown [16]. However, often, an inverse relationship between these pathways is observed. In this regard, anti-inflammatory agents that suppress NF-κB signaling may activate Nrf2. In addition, Nrf2 may reverse the cellular damage of inflammation-exposed cells and indirectly prevent the sustained activation of NF-κB [16].

Apart from NF-κB and Nrf2, several other signaling pathways might be involved in inflammation and, thus, pancreatic cancer induction. These include MAPKs and the Jak/STAT, CREB, and PI3K/Akt pathways [17]. In this regard, up to 60% of pancreatic cancer tissues and most pancreatic cancer cell lines exhibit increased Akt activity [18]. Moreover, crosstalk between these pathways is possible [17]. Therefore, suppressing multiple signaling pathways involved in inflammation responses may be a promising approach for the treatment of pancreatic cancer. 

Several studies have shown that the use of nonsteroidal anti-inflammatory drugs (NSAIDs) is associated with decreased cancer incidence and recurrence. The specific COX-2 inhibitor, celecoxib, was approved by the FDA for use in the adjuvant therapy of familial adenomatous polyposis [19]. Beneficial effects after treatment with several COXIBs, e.g., celecoxib and 2,5-dimethylcelecoxib, were also observed in glioblastoma cells [20]. Moreover, early studies showed that long-term use (>2 years) of NSAIDs may protect against pancreatic cancer, but replication of these findings was recommended [21]. However, in general, anti-inflammatory agents, even those that obtained FDA approval, have limited use in cancer prophylaxis or therapy because of the presence of off-target effects and toxicities [19]. Therefore, it is important to develop safe and effective agents that reduce cancer risk. Coupling NSAIDs with natural compounds exhibiting anti-inflammatory potential might enhance their anti-inflammatory activity and prevent the unfavorable side effects of the long-term use of NSAIDs. One such compound is the naturally occurring triterpenoid oleanolic acid (OA) and its synthetic derivatives. Our earlier studies showed that conjugates of NSAIDs, namely, aspirin, indomethacin (IND), or diclofenac (DCL) with novel synthetic oxime derivatives of OA (OAO) enhanced their modulating effect on Nrf2-ARE and NF-κB signaling pathways in hepatoma cancer cells (HCC), resulting in increased apoptosis rate and reduced cell proliferation. Moreover, the DCL hybrid with OAO morpholide derivative reduced the tumor volume in mice bearing xenografts [22]. 

**Figure 1:**
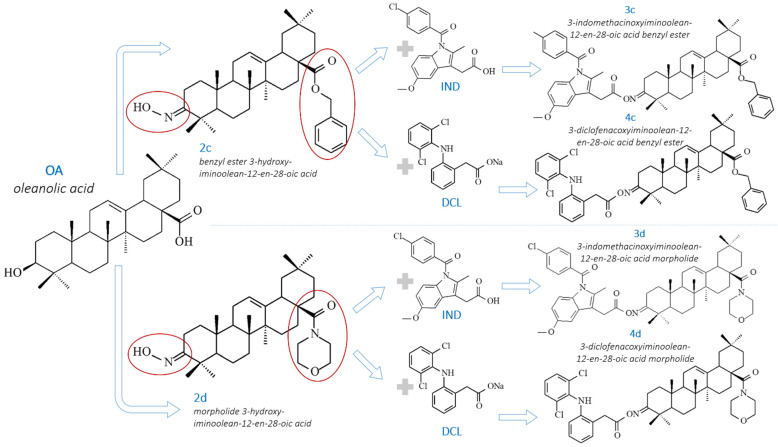
The chemical structures of oleanolic acid (OA), oleanolic acid oximes derivatives: benzyl ester, and morpholide (2c, 2d), and their conjugates with IND (3c, 3d) and DCL (4c, 4d).

The aim of our present study was to evaluate the effect of the IND and DCL conjugates (3c, 3d, 4c, 4d) with two OAO derivatives, namely, benzyl ester and morpholide (2c, 2d) (Figure 1), on the signaling pathways involved in the inflammation process in pancreatic cancer cells, and their possible effects on cell cycle distribution and proliferation.

## 2. Results

### 2.1. Conjugation with OAO Derivatives Increases the Cytotoxicity of Indomethacin and Diclofenac

**Figure 2:**
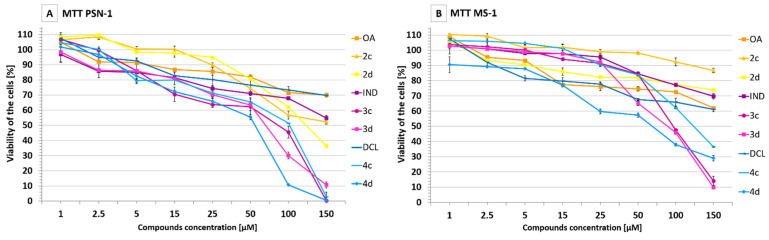
The effect of the OA, OAO derivatives (2c, 2d), and OAO conjugates with IND (3c, 3d) and DCL (4c, 4d) on the viability of PSN-1 (**A**) and MS-1 cells (**B**). Data are expressed as means ± SEM from three separate measurements.

The viability of pancreatic adenocarcinoma-derived PSN-1 cells and immortalized pancreatic endothelial cells MS-1 was assessed after treatment with OA, OAO, DCL, IND, and their conjugates at the concentration range of 1–150 µM. As shown in Figure 2A,B, and Table 1, the conjugation of IND and DCL with OAO significantly increased their cytotoxicity, reaching even 100% in PSN-1 cells at the highest tested concentration. Comparing the IC_50_ values obtained on both cell lines, slightly higher cytotoxicity toward the cancer PSN-1 cells was noticed.

Based on the MTT assay results, the concentrations of 30 µM assuring 70% viability were applied in the further assays.

### 2.2. IND and DCL-OAO Conjugates Diminish the Activation of NF-κB and COX-2 Expression in Pancreatic Cancer PSN-1 Cells

The active NF-κB generally refers to a p50-p65 heterodimer, depicting the major Rel/NF-κB complex in cells [23]. Translocation of the active heterodimer into the nucleus, where it binds with specific sequences of the target genes, results from phosphorylation of the IκB by the IκB kinase beta (IKK), leading to a degradation of the inhibitory subunit [24]. Therefore, the effect of OAO and their conjugates with IND and DCL was assessed, based on the binding of their p50 and p65 subunits to their immobilized consensus site and their nuclear level. 

**Figure 3:**
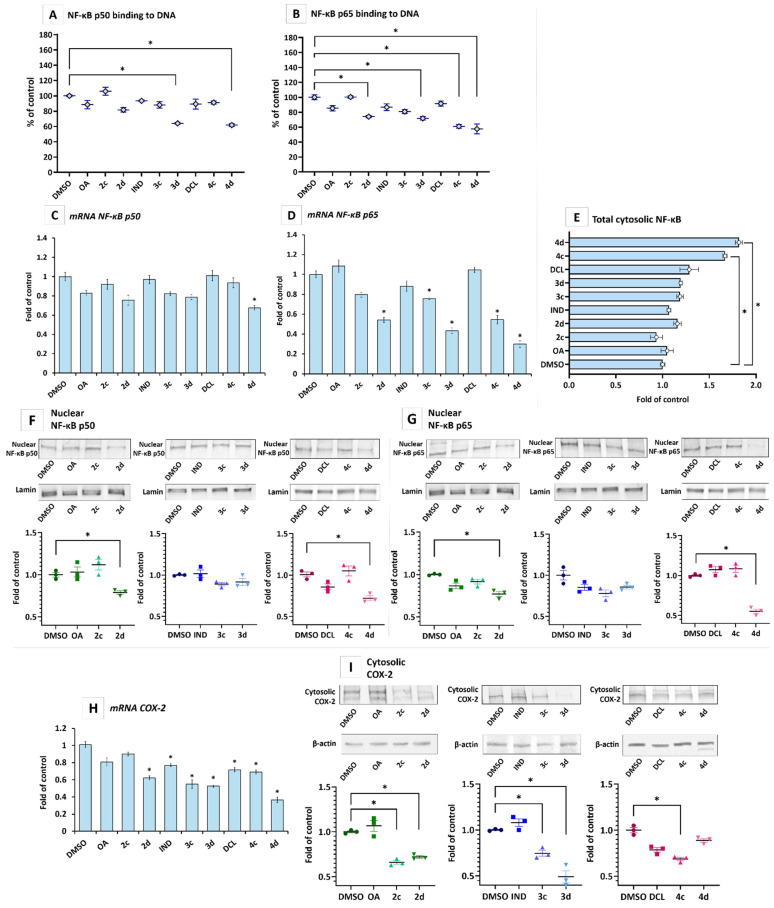
The effect of the OA, OAO derivatives (2c, 2d), and OAO conjugates with IND (3c, 3d) and DCL (4c, 4d) in the PSN-1 cells treated with these compounds at concentrations of 30 µM for 24h on NF-κB activation and expression. Panels (**A**,**B**) show NF-κB p50 and p65 subunits binding to DNA, its cytosolic and nuclear protein levels panels (**E**–**G**) and mRNA transcripts panels (**C**,**D**). Panels (**H**,**I**) show the COX-2 gene mRNA transcript and protein. Activated p50 and p65 subunits were assessed regarding the amount of NF-κB p50 (**A**) and NF-κB p65 (**B**) contained in the DNA-binding complexes extracted from the nuclear fraction. The values (mean ± SEM) from three separate experiments are presented in comparison to control cells, set to 100%. Representative Western immunoblots of the nuclear NF-κB p50 (**F**), NF-κB p65 (**G**), and cytosolic COX-2 (**I**) are shown above the graphs. The sequence of the bands corresponds to the sequence of the bars in the graph. Lamin and β-actin were used as a loading control. The values were calculated as protein levels compared to control cells (expression equal to 1). The total cytosolic NF-κB protein level (**E**) was assessed using the MAGPIX^®^ system, and the results from three independent measurements are presented as a fold of fluorescence intensity obtained in control cells. The values (mean ± SEM) of mRNA levels (**C**,**D**,**H**) were calculated as the relative change in comparison to control cells (expression equal to 1). * Significantly different from control: DMSO-treated cells, *p* < 0.05. Student’s *t*-test was used to assess the statistical significance.

As shown in Figure 3A,B, hybrids 3d and 4d, i.e., conjugates of IND and DCL with OAO morpholide, were the most efficient at reducing the binding level of p50 and p65. However, the effect was more pronounced for the p65 subunit. The latter was also reduced by the conjugate of DCL with OAO benzyl derivative (4c) and OAO morpholide itself (2d). The reduced binding of the p65 subunit to its consensus site after treatment with compounds 4d and 2d correlated with the level of p65 nuclear protein (Figure 3F) and total cytosolic protein (Figure 3E).

The reduced activation of NF-κB in the case of the p65 subunit might also be related to a diminished expression of its gene, as indicated by the reduced level of p65 mRNA (Figure 3D) after treatment with compounds 4c and 4d, 3c and 3d and OAO morpholide (2d). The 4d derivative caused ~60% diminished expression of p65 mRNA, while the expression of p50 was affected only by this derivative, although it merely caused a ~30% decrease (Figure 3C). One of the NF-κB target genes is COX-2, which similarly to NF-κB, is often overexpressed in pancreatic cancer cells [25]. Thus, as a result of the diminished activation and expression of NF-κB, reduced COX-2 transcript and protein levels occurred as a result of treatment with compounds 2c, 2d, 3c, 3d, and 4c (Figure 3H,I). Hybrid 4d significantly reduced the COX-2 transcript level but did not affect its protein level.

These data indicate that IND and DCL conjugates with OAO morpholide are the most effective inhibitors of NF-ĸB in pancreatic cancer cells, unlike IND and DCL and the OAO derivatives, which on their own showed a negligible or no effect.

### 2.3. Conjugates with OAO Increased Activation and Expression of Nrf2 in PSN-1 Cells

**Figure 4:**
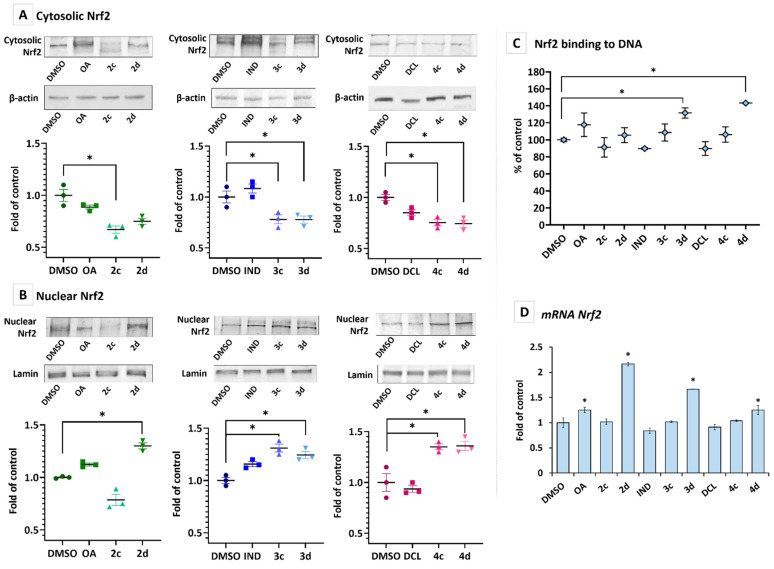
The effect of the OA, OAO derivatives (2c, 2d), and OAO conjugates with IND (3c, 3d) and DCL (4c, 4d) in the PSN-1 cells treated with these compounds at concentrations of 30 µM for 24 h on Nrf2 activation and expression. Panels (**A**,**B**) present cytosolic and nuclear protein levels of Nrf2, panel (**C**) Nrf2 binding to DNA, and mRNA transcript panels (**D**). Representative Western immunoblots are presented above the graphs. The sequence of the bands corresponds to the sequence of the bars in the graph. Lamin and β-actin were used as a loading control. The values were calculated as protein levels compared to control cells (expression equal to 1). Activated Nrf2 (**C**) was assessed in terms of the amount of Nrf2 contained in the DNA-binding complexes extracted from the nuclei isolated from the cells and calculated compared to control cells set to 100%. The values (means ± SEM) of mRNA level (**D**) were calculated as the relative change in comparison to control cells (expression equal to 1). * Significantly different from control: DMSO-treated cells, *p* < 0.05. Student’s *t*-test was used to assess the statistical significance.

The activation of Nrf2 requires its translocation from the cytosol into the nucleus. As shown in Figure 4A,B, treatment with the DCL conjugate with OAO benzyl ester (4c) and morpholide (4d) significantly increased the Nrf2 translocation and its level in the nucleus. The effect of the same conjugates with IND (3c, 3d) was slightly less pronounced. An increased level of Nrf2 in the nucleus resulted in its enhanced binding to the ARE sequence, measured based on the amount of Nrf2 contained in the DNA binding complex to the ARE sequence. The oligonucleotides containing ARE consensus-binding site (5′-GTCACAGTGACTCAGCAGAATCTG-3’) for Nrf2 were immobilized on microplates as bait (Figure 4C). An increased level of Nrf2 transcript was observed after treatment with OAO morpholide and its conjugates with IND and DCL. Increased levels of Nrf2 transcript were also observed after treatment with OA and OAO morpholide (Figure 4D).

The activation of Nrf2 leads to the elevated mRNA levels of its target genes *SOD-1*, *NQO1*, and *GSTA* encoding superoxide dismutase, NAD(P)H: quinone oxidoreductase, and glutathione-S-transferase A, respectively. 

**Figure 5:**
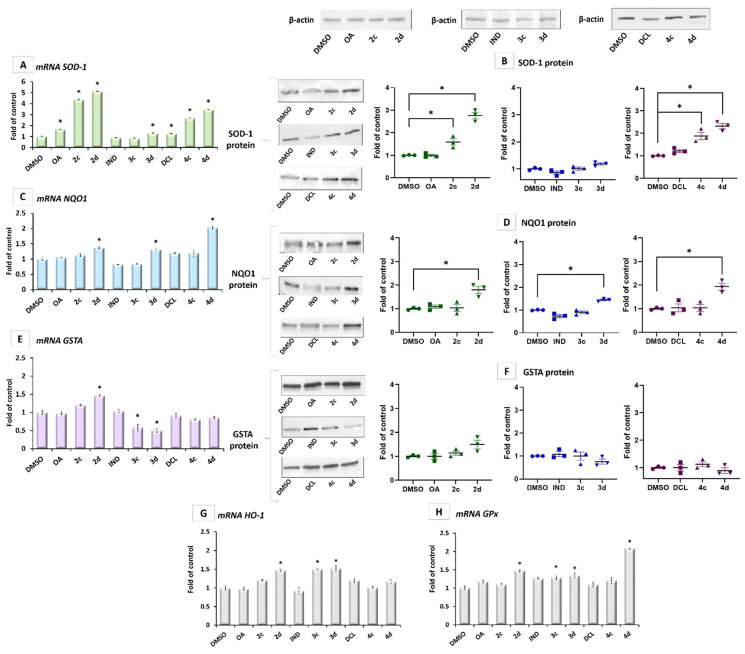
The effect of the OA, OAO derivatives (2c, 2d), and OAO conjugates with IND (3c, 3d) and DCL (4c, 4d) on the SOD-1 (**A**,**B**), NQO1 (**C**,**D**), and GSTA (**E**,**F**) mRNA expression and protein levels, as well as HO-1 (**G**) and GPx (**H**) mRNA expression levels, measured in PSN-1 cells after 24 h incubation with tested compounds at the concentrations of 30 µM. The values (mean ± SEM) for the mRNA expression levels (**A**,**C**,**E**,**G**,**H**) were calculated from three separate experiments in comparison with control cells (expression of control was set as 1). Representative immunoblots for the analysis of the cytosolic levels of SOD-1 (**B**), NQO1 (**D**), and GSTA (**F**) are shown. The sequence of the bands corresponds to the sequence of the bars in the graph. β-actin was used as a loading control. The values were calculated as protein levels compared to control cells (expression equal to 1). * Significantly different from control: DMSO-treated cells, *p* < 0.05. Student’s *t*-test was used to assess the statistical significance.

While this increased transcript level after treatment with 4c and 4d was confirmed for SOD-1 protein level (Figure 5A,B) and, in the case of 4d, on NQO1 protein (Figure 5C,D), those conjugates did not affect the GSTA protein (Figure 5F). In contrast, conjugates 3c and 3d even showed a tendency to reduce the expression of this gene (Figure 5E). Since increased expression of GSTA is often associated with an increased risk of cancer, this effect might be considered to be desirable [26]. The transcript of *HO-1* and *GPx* genes encoding heme oxygenase-1 and glutathione peroxidase was elevated by about 30–50% after treatment with 2d, 3c, and 3d derivatives (Figure 5G,H). DCL conjugate with OAO morpholide derivative was the most potent inducer of the GPx gene, leading to a more than a two-fold increase in its transcript level (Figure 5H). 

These results show that conjugates of OAO derivatives, particularly OAO morpholide with DCL, in concert with inhibition of the NF-κB pathway, activate Nrf2 signaling, leading to the increased expression of antioxidant enzymes.

**Figure 6:**
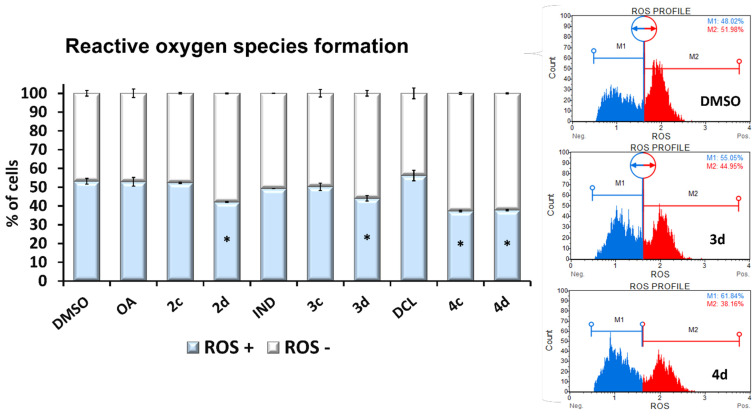
The effect of the OA, OAO derivatives (2c, 2d), and OAO conjugates with IND (3c, 3d) and DCL (4c, 4d) on the generation of reactive oxygen species in PSN-1 cells after 24 h incubation with tested compounds at concentrations of 30 µM. Graphs represent the relative percentage of cells that are ROS-negative and -positive (percentage of cells undergoing oxidative stress, based on the intracellular detection of superoxide radicals). The mean values (mean ± SEM) from three independent experiments are shown. * Significantly different from control: DMSO-treated cells, *p* < 0.05. Student’s *t*-test was used to assess the statistical significance.

The increased expression of SOD-1 may be the reason for the observed reduced production of ROS (namely, superoxide radicals) as a result of treatment with 2d, 3d, 4c, and 4d (Figure 6).

### 2.4. Bead-Based Multiplex Immunoassay Revealed Possible Modulation of Protein Regulating Several Signaling Pathways by OAO-IND and OAO-DCL Conjugates

**Figure 7:**
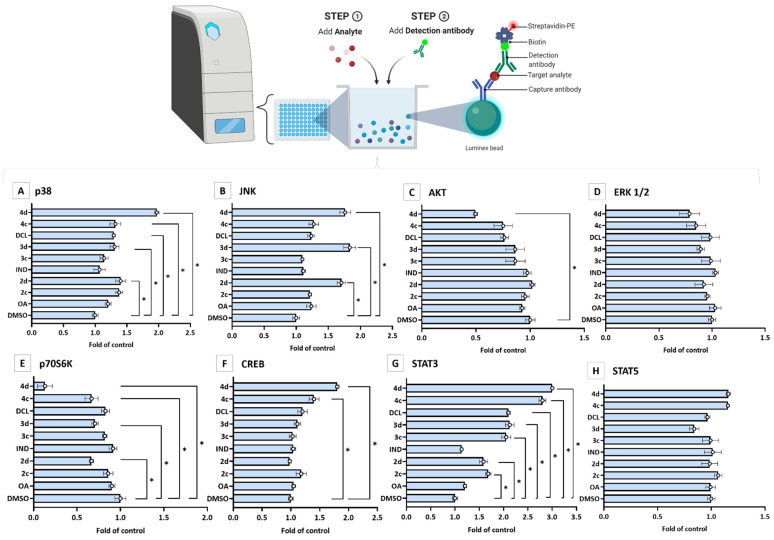
The effect of the OA, OAO derivatives (2c, 2d), and OAO conjugates with IND (3c, 3d) and DCL (4c, 4d) on the cytosolic level of p38 (**A**), JNK (**B**), AKT (**C**), ERK1/2 (**D**), p70S6K (**E**), CREB (**F**), STAT3 (**G**) and STAT5 (**H**), measured and calculated on the MAGPIX^®^ system in PSN-1 cells, after 24 h incubation with tested compounds at concentrations of 30 µM. The mean values (mean± SEM) of fluorescence intensity are presented as the fold of control from three independent measurements. * Significantly different from control: DMSO-treated cells, *p* < 0.05. Student’s *t*-test was used to assess the statistical significance.

A bead-based multiplex immunoassay was applied to evaluate the effect of tested compounds on cytosolic levels of kinases from the MAPK family, p38, JNK, AKT, ERK 1/2, and p70S6K. Additionally, CREB, STAT3, and STAT5 protein levels were assessed. As shown in Figure 7, significant decreases of cytosolic AKT and p70S6K protein levels, as a result of treatment with 4d, were observed, reduced by ~50% and ~90%, respectively. In contrast, the same compound most efficiently increased the levels of certain proteins: CREB (by ~80%), JNK (by ~70%), and STAT3 (three-fold increase). ERK1/2 and STAT5 were relatively unaffected. A decreased level of p70S6K was observed as a result of treatment with compounds 4c, 3d, and 2d, while an increase in the p38 protein level was detected after treatment with 4c, DCL, 3d, and 2d. STAT3 protein level was affected by all tested compounds except OA and IND. 

### 2.5. Conjugates of IND, DCL, and OAO Derivatives Do Not Change the Cell Cycle Distribution and Have No Effect on Cell Proliferation in Pancreatic Cancer Cells

**Figure 8:**
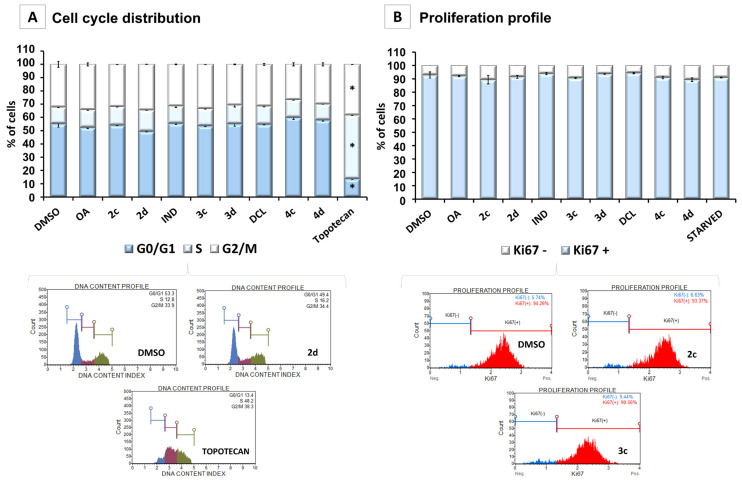
The effect of the OA, OAO derivatives (2c, 2d), and OAO conjugates with IND (3c, 3d) and DCL (4c, 4d) on the cell cycle distribution (**A**) and the proliferation level (**B**) measured in PSN-1 cells after 24 h incubation with tested compounds at concentrations of 30 µM. (**A**) Graphs represent the percentage of cells in the G1/G0, S, and G2/M phases measured by flow cytometry after propidium iodide staining. Topotecan (100 nM)-treated cells were used as a positive control for the cell cycle arrest. (**B**) Graphs representing the percentage of proliferating [Ki67(+)] and nonproliferating [Ki67(−)] cells measured by flow cytometry. Starved cells (cultured in an FBS-free medium) were used as a reference for the antiproliferative conditions. Representative plots are presented. The mean values ± SEM from three independent experiments are shown for both panels. * Significantly different from control: DMSO-treated cells, *p* < 0.05. Student’s *t*-test was used to assess the statistical significance.

Figure 8A shows the effect of OA, its oximes derivatives, and their conjugates with IND or DCL on cell cycle distribution in the PSN-1 cells. While topotecan, used as a reference compound, significantly increased the percentage of the cells in the S phase and G2/M, none of the tested compounds changed the cell-cycle distribution. The effect of the tested OA derivatives and conjugates on cell proliferation was evaluated, based on Ki67 protein expression. Similar to effects on cell cycle distribution, the compounds did not affect PSN-1 cell proliferation (Figure 8B). Although the reduced activation of NF-κB may enhance proapoptotic gene expression, the enhanced activation of Nrf2 and reduced ROS production may be responsible for the lack of induction of cell cycle arrest or inhibition of cell proliferation by the tested compounds. This concept supports the fact that the proliferation and differentiation of diverse cell types are often influenced and modulated by the cellular redox balance. Therefore, Nrf2 regulating the cellular levels of ROS may control these cellular processes. 

## 3. Discussion

Chronic inflammation is considered one of the major risk factors of PC. During chronic pancreatitis, inflammatory cells and macrophages enhance stroma formation, which increases the risk of PC development. Moreover, enhanced activation of the NF-κB signaling pathway, in concert with an increased level of cytokines and chemokines, was observed in this type of cancer [27]. Thus, the NF-κB pathway is emerging as an important element in PC cancer development [28]. Consequently, NSAIDs, along with natural compounds with anti-inflammatory potential, might be considered as therapeutic agents targeting this signaling pathway.

Our earlier studies showed that conjugates of OAO derivatives, particularly morpholides and benzyl esters with ASP [29,30], IND [31], or DCL [22], effectively inhibited NF-κB activation and expression in HCC cells, resulting in a reduced rate of proliferation. The focus of this study was to evaluate whether the same effect might be observed in pancreatic cancer cells. 

While the highest activity of IND and DCL conjugates with OAO morpholide was confirmed, this effect was generally less pronounced in PSN-1 cells than in HCC cells, requiring higher concentrations of the compound. Initial screening of the effects of these compounds, i.e., their impact on the PSN-1 cells at the concentrations applied in HCC cells (10 and 20 µM), in most cases, did not show significant effects. Therefore, a concentration of 30 µM was used in further experiments. At this dose, significant changes in the activation and expression of NF-κB and related signaling pathways were observed. The activation of NF-κB was measured in terms of binding its active subunits, p65 and p50, to DNA, and their nuclear levels were the most reduced as a result of treating PSN-1 cells with OAO-DCL morpholide hybrid (4d), although the conjugate of DCL with OAO-benzyl ester (4c) as well as OAO-morpholide itself (2d) were also effective inhibitors of this pathway. These derivatives also affected NF-κB expression, as indicated by the reduced level of p65 transcript, while p50 mRNA was diminished only by OAO benzyl and morpholide derivatives.

The effect of tested compounds, similar to our previous study in HCC cells, was more explicit for the p65 NF-κB subunit than for p50. The p65 subunit is basically responsible for the transcription initiation, while the p50 subunit serves only as a helper in NF-κB DNA binding [32,33]. Excessively activated p65 subunit and, ultimately, effector molecules might be related to PC pathogenesis [5]. One such effector molecule, often overexpressed in PC, is COX-2 [25]. All compounds tested in this study, except OA and 2c, reduced the expression of *COX-2* at the mRNA level, but this effect was not confirmed for the protein level in the case of conjugate 4d, which reduced COX-2 mRNA to the greatest extent. There are many processes between transcription and translation that affect the protein level and contribute to this discrepancy. The half-life of different proteins can vary from minutes to days, whereas the degradation rate of mRNA would fall within a much tighter range of 2–7 h for mammalian mRNAs, vs. 48 h for protein [34].

NF-κB negatively interferes with the Nrf2 signaling pathway, and, often, agents that suppress NF-κB signaling will activate Nrf2 [35]. The activity of Nrf2 is regulated by the Keap1 protein, which promotes the ubiquitination and subsequent degradation of Nrf2 under normal conditions while releasing the active Nrf2 upon exposure to stress [36]. The activation of protein kinases, such as extracellular signal-regulated kinase (ERK), induces Nrf2 phosphorylation, which may also stimulate the dissociation of Nrf2 from its repressor Keap1 and its subsequent translocation into the nucleus and binding to the ARE sequence [37]. This study showed enhanced activation of the Nrf2 pathway, as indicated by the translocation of its active form into the nucleus and binding to the ARE sequence, particularly by treatment with IND and DCL hybrids with OAO morpholide (3d, 4d) in PSN-1 cells. These compounds, along with OAO morpholide (2d) itself, also increased Nrf2 expression at the mRNA level. The same effect in the case of OAO morpholide and the other OAO derivatives was observed in human hepatoma HepG2 cells. However, in HCC cells, it was less pronounced as a result of treatment with their conjugates with ASP [29]. Moreover, the association between cytotoxicity and Nrf2 activation was observed in these cells, i.e., the most cytotoxic derivatives activated Nrf2 to the greatest extent [26,27]. Basically, the same effect was observed in this study in pancreatic cancer cells. 

The contribution of the Nrf2-ARE signaling pathway to protection against reactive oxygen species (ROS) and electrophilic insults has been well established and occurs through the induction of genes encoding antioxidant enzymes. Activation of Nrf2 stimulated by OAO derivatives 2c and 2d in PSN-1 cells increased the expression of SOD-1, GPx, HO-1, and NQO1 genes, similar to the effects seen in HepG2 cells.

However, in contrast to a conjugate with ASP, a hybrid of DCL and, to a lesser extent, the IND hybrid were the most potent inducers of these genes. The proteins encoded by these genes play an important role in protecting against reactive electrophiles produced as a result of xenobiotic exposure and ROS, which are involved in PC pathogenesis [38]. Consequently, reduced production of ROS under the influence of these compounds was found. In this way, enhanced activation of Nrf2 may play protective roles in pancreatic inflammation. Thus, the tested compounds might be considered chemopreventive agents. On the other hand, accumulating evidence suggests that an amplified activity of Nrf2 may promote cancer growth and increase chemoresistance [39]. In this regard, constitutive overexpression of Nrf2 in PC models such as the K-Ras (G12D), B-Raf (V619E), Myc (ERT2), and human samples was described [16]. 

However, it was demonstrated using synthetic triterpenoid RTA 405 as an example that the pharmacological activation of Nrf2 is distinct from genetic activation and does not provide a growth or survival advantage to several cancer cells, including PC cells. Moreover, it was shown that pretreatment with RTA 405 did not protect cancer cells from doxorubicin- or cisplatin-mediated growth inhibition [40].

MAPKs such as ERK, JNK, and p38 are key molecules at the convergence of several signal transduction pathways. They transduce the converged signals into the nucleus, resulting in various cellular responses, including proliferation, differentiation, and apoptosis [41]. Several studies have correlated the phosphorylation of ERK and Nrf2 activation [42]. Interestingly, it was shown that the activation of MAPK–Akt and ERK is required for OA-induced activation of Nrf2, followed by HO-1 expression, in primary rat vascular smooth muscle cells [43]. Using a bead-based multiplex immunoassay in our study, we observed decreased levels of cytosolic AKT and increased levels of unphosphorylated JNK and p38 [44,45]. The accumulation of p38 and JNK in the cytosol could result from decreased ROS production and may be related to an increase in cytosolic CREB protein level [46]. The latter is often overexpressed and constitutively phosphorylated in many types of cancer, including pancreatic cancer [47]. Therefore, its reduced activation is desirable. Additionally, increased STAT3 cytosolic level may suggest the inhibition of mTOR signaling as the crosstalk between these pathways has been implicated in several malignant diseases, e.g., osteosarcoma [48]. Moreover, a reduced level of cytosolic p70S6 kinase, which is activated by mTOR, further supports this concept [49]. 

Overall, our current study indicates that the conjugation of NSAIDs with OAO derivatives may enhance their modifying effect on key signaling pathways, not only in HCC but also in PC cells. Moreover, in the PC cells, the conjugation of DCL with OAO morpholide (4d) shows greater efficiency than OAO morpholide (2d) alone. However, in contrast to HCC cells, reduced activation of NF-κB led to enhanced activation of Nrf2, which may be responsible for the lack of effect on cell-cycle distribution and proliferation. Such an interpretation of the data presented in this study is further supported by the fact that the analyses of the genome-wide distribution of Nrf2 have identified new sets of Nrf2 target genes whose products are indeed involved in cell proliferation and differentiation. Moreover, it was shown that Nrf2 is required for the transition from the G2 phase to the M phase in the cell cycle [50].

Importantly, IND or DCL alone did not affect the parameters evaluated in PC cells. Since inflammation can predispose to PC, NSAIDs were proposed for cancer prevention and therapy [19,21]. However, the output of these attempts is not consistent. Therefore, the results of this study, indicating that DCL conjugate with OAO morpholide is a more potent modulator of the key signaling pathways in PC cells than parent compounds, may pave the way to designing more intensive in vitro and subsequently in vivo studies on these types of hybrids for prophylaxis and/or PC treatment. 

## 4. Materials and Methods

### 4.1. Chemistry

OAO derivatives (2c, 2d) and OAO conjugates with IND (3c, 3d) and DCL (4c, 4d) were synthesized as described previously [22,30,31]. Their chemical structures were elucidated based on IR, ^1^H NMR, ^13^C NMR, MS, and HRMS elemental analysis. The NMR spectra and the detailed description of synthesized compounds’ identity were presented in our previous papers, along with the synthesis overview and detailed purity data.

### 4.2. Biological Assays

#### 4.2.1. Cell Culture and Viability Assay

Pancreatic carcinoma cell lines, PSN-1, and immortalized pancreatic MS-1 cell lines were purchased from American Type Culture Collection (Rockville, MD, USA). The PSN-1 cells were maintained in RPMI-1640 medium (Sigma-Aldrich, St. Louis, MO, USA) and MS-1 cells in Dulbecco’s Modified Eagle’s Medium (Sigma-Aldrich, St. Louis, MO, USA). Both cell lines were maintained with 10% FBS (EURx, Gdańsk, Poland), 1% antibiotic solution (Sigma-Aldrich, St. Louis, MO, USA), and were grown under standard conditions (37 °C and 5% CO_2_). After 24 h of initial incubation, the cells were treated with 0.1% of vehicle control and tested compounds at a concentration of 30 µM for a further 24 h and then harvested. The concentration of OAO derivatives and their conjugates was selected based on the MTT viability assay.

The MTT assay was performed following the standard protocol. Briefly, PSN-1 and MS-1 cells were seeded at 10^4^ cells per well in 96-well plates. After 24 h of preincubation in a complete medium, compounds were added, and cells were incubated for 24 h. Later, the cells were washed twice with phosphate-buffered saline (PBS) and incubated for 4 h with a medium containing 0.5 mg/mL 3-(4,5-dimethylthiazol-2-yl)-2,5-diphenyl-2H-tetrazolium bromide (MTT). Then, the formazan crystals were dissolved in acidic isopropanol, and the absorbance was measured at 570 nm and 690 nm. All experiments were repeated three times.

#### 4.2.2. Total RNA Isolation, cDNA Synthesis, and Quantitative Real-Time PCR (R-T PCR)

The total RNA extraction was performed using the GeneMatrix Universal DNA/RNA/Protein Purification Kit (EURx, Gdańsk, Poland). According to the manufacturer’s instructions, samples were subjected to reverse transcription via the RevertAid First Strand cDNA Synthesis Kit (Thermo Fisher Scientific, Waltham, MA, USA).

The Maxima SYBR Green Kit (Thermo Fisher Scientific, Waltham, MA, USA) and the BioRad Chromo4 thermal cycler (BioRad Laboratories, Hercules, CA, USA) were used for the quantitative R-T PCR analyses. Protocol started with 5 min enzyme activation at 95 °C, followed by 40 cycles of 95 °C for 15 s, 56 °C for 20 s and 72 °C for 40 s, and the final elongation at 72 °C for 5 min. Melting curve analysis was used for amplicon verification. The expression of *TBP* (*TATA box binding protein*) and *PBGD* (*porphobilinogen deaminase*) was used to normalize data. The Pfaffl comparative method was used for fold-change quantification. Primers were designed using the Beacon Designer software and subjected to a BLAST search to minimize unspecific binding. Only the primer pairs that generated intron-spanning amplicons were selected. The primers’ sequences used to analyze *NF-кB p50, NF-кB p65, COX-2, Nrf2, SOD-1, NQO1, GSTA, HO-1, GPx, PBGD,* and *TBP* genes are listed in Table 2. 

#### 4.2.3. Nuclear and Cytosolic Fractions Preparation

According to the manufacturer’s protocol, the subcellular extracts from PSN-1 cells were prepared using the Nuclear/Cytosol Fractionation Kit (BioVision Research, Milpitas, CA, USA). Protein concentration was assessed, and the samples were stored at −80 °C for further analysis.

#### 4.2.4. Western Blot Analysis 

Cytosolic extracts for Nrf2, SOD-1, NQO1, GSTA, COX-2, and β-actin, or nuclear extracts for Nrf2, NF-кB p65, NF-кB p50, and lamin protein detection, were separated on 12% or 10% SDS-PAGE slab gels. The β-actin and lamin were used as a loading control. Then, 100 µg of cytosolic and nuclear fractions were added per well for the SDS-PAGE. Proteins were transferred to the nitrocellulose Immobilon P membrane. After blocking for 2 h with 10% skimmed milk, proteins were probed with primary antibodies against Nrf2, NF-кB p65, NF-кB p50, SOD-1, NQO1, GSTA, COX-2, β-actin, and lamin. Alkaline phosphatase AP-labeled anti-rabbit IgG, anti-goat IgG, and anti-mouse IgG secondary antibodies (BioRad Laboratories, Hercules, CA, USA) were used in the staining reaction. Bands were visualized with the AP Conjugate Substrate Kit NBT/BCIP (BioRad Laboratories, USA). The number of immunoreactive products in each lane was determined using the ChemiDoc Imaging System (BioRad Laboratories, Hercules, CA, USA). Values were calculated as relative absorbance units (RQ) per mg of protein and expressed as a percentage of control.

#### 4.2.5. Nrf2 and NF-ĸB Binding Assay 

Nrf2, NF-κB p50, and NF-κB p65 activation were assessed by the enzymatic immunoassay (Transcription Factor ELISA Assay Kit; Active Motif, La Hulpe, Belgium) according to the manufacturer’s instructions. The activated Nrf2 was evaluated based on the amount of Nrf2 contained in the DNA-binding complex to the ARE sequence. The oligonucleotides containing the ARE consensus-binding site (5′-GTCACAGTGACTCAGCAGAATCTG-3′) for Nrf2 were immobilized on microplates as bait. However, the activated NF-ĸB was measured using the number of p65 and p50 subunits held in the DNA-binding complex. The oligonucleotides containing (5’-GGGACTTTCC-3’), a consensus site for NF-κB, were immobilized on microplates as bait. Nuclear fractions were incubated with oligonucleotides for 1 h, then the wells were washed, and DNA-bound subunits were detected by the specific primary antibody and a secondary antibody conjugated with the HRP. The results were expressed as the normalized level of absorbance (OD450 nm per mg of protein).

#### 4.2.6. Cell Cycle Distribution

The analysis of the cell cycle distribution was performed using the Muse Cell Cycle Kit (Merck, Darmstadt, Germany) according to the manufacturer’s protocol. Briefly, cells (3 × 10^5^ per well) were seeded and grown on 6-well plates for 24 h before stimulation. Then, the tested compounds were added and cells were incubated for 24 h. Topotecan (100 nM)-treated cells were used as a positive control for the cell cycle arrest. Subsequently, cells were harvested by trypsinization, washed with PBS buffer, fixed in ice-cold 70% ethanol, and frozen until staining at −20 °C. After overnight storage, cells were washed with cold PBS buffer, stained with propidium iodide in the presence of RNAase A, and, after 30 min incubation at room temperature and in the dark, the fluorescence signal was analyzed by flow cytometry on the Muse Cell Analyzer (Merck, Darmstadt, Germany). Data were evaluated using Muse 1.5 analysis software (Merck, Darmstadt, Germany).

#### 4.2.7. Proliferation Profile

According to the manufacturer’s protocol, the Ki67 protein was used as a marker of proliferating cells, and its expression was detected by fluorochrome-labeled antibody using the Muse Ki67 Proliferation Kit (Merck, Darmstadt, Germany). Briefly, cells (3 × 10^5^ per well) were seeded on the 6-well plates. After 24 h, the tested compounds were added, and cells were then incubated for 24 h. Cells cultured in a medium without FBS (starved cells) were used as a positive control of antiproliferative conditions. After the incubation, cells were collected by trypsinization, washed with PBS buffer, instantly fixed, and exposed to a permeabilization buffer. After 30 min incubation at room temperature and dark with the Muse Hu Ki67-PE antibody, cells were analyzed on Muse Cell Analyzer (Merck, Darmstadt, Germany) by flow cytometry. Data were evaluated using Muse 1.5 Analysis Software (Merck, Darmstadt, Germany).

#### 4.2.8. ROS Generation

Dihydroethidium (DHE) reacts with superoxide radicals, generating fluorophores with a high affinity to DNA. The Muse Oxidative Stress Kit (Merck, Darmstadt, Germany) containing DHE was used according to the manufacturer’s protocol for the quantitative measurement of superoxide radical positive cells. Briefly, cells (3 × 10^5^ per well) were seeded in the 6-well plates. After 24 h, the tested compounds were added, and cells were incubated for 24 h. Subsequently, cells were collected by trypsinization, washed with PBS buffer, resuspended in 1X Assay Buffer, mixed with Muse Oxidative Stress Reagent working solution, and subjected to a 30 min incubation (37 °C, 95% humidity, 5% CO_2_). Cells were analyzed by flow cytometry on the Muse Cell Analyzer (Merck, Darmstadt, Germany), and data were evaluated using the Muse 1.5 Analysis Software (Merck, Darmstadt, Germany).

#### 4.2.9. Bead-Based Immunoassay on the Luminex MAGPIX Instrument

A magnetic bead-based immunoassay was performed on the Luminex-MAGPIX multiplex immunoassay system. Data were analyzed with MILLIPLEX^®^ Analyst 5.1 software (EMD Millipore, Burlington, MA, USA). The panel that we performed quantitated the following proteins in the cytosolic fractions of PSN-1 cells, NF-κB, AKT, p70S6, p38, JNK, ERK 1/2, CREB, STAT3, and STAT5, according to the manufacturer’s instructions. The magnetic bead panel, with high-sensitivity antibodies, was obtained from Merck (Darmstadt, Germany). A multiplex test based on microspheres, using Luminex xMAP technology with different fluorescent colors, was detected on a compatible MAGPIX camera. Cytosolic fractions were suspended in MILLIPLEX^®^ MAP buffer. The bead suspension was added to each well of a 96-well plate, then samples were added into the wells and incubated overnight at 2–8 °C on a shaker protected from light. The plate was washed with 2× buffer, and then 1X MILLIPLEX^®^ MAP detection antibodies were added. After shaking for 1 h at room temperature, the antibodies were removed, and 1X MILLIPLEX^®^ MAP Streptavidin/Phycoerythrin (SAPE) was added. Then the MILLIPLEX^®^ MAP Amplification Buffer was added to each well and shaken for 15 min. The beads were suspended in MILLIPLEX^®^ MAP buffer, and each microsphere was identified with a MAGPIX Luminex Analyzer (Merck, Darmstadt, Germany); the results were calculated from the reporters’ fluorescent signals. Mean fluorescence intensities were quantified with the xPonent 4.2 software (Luminex Corporation, Austin, TX, USA). The raw MFI results for the tested protein levels were converted, relative to the control of DMSO-treated cells.

### 4.3. Statistical Analysis

Statistical analysis and graphs were calculated and prepared using GraphPad Prism 9.2.0 (GraphPad Software, San Diego, CA, USA), assuming the significance level of changes as *p* < 0.05. Student’s *t*-test was used to assess the statistical significance between the experimental and control groups.

## 5. Conclusions

We show here that the conjugation of NSAIDs with OAO derivatives enhances their effect on key signaling pathways that are involved in the inflammation process. Our studies indicating that DCL conjugate with OAO morpholide is a more potent modulator of the key signaling pathways in PC cells than parent compounds should pave the way to the design of more intensive studies on these types of hybrids for PC treatment and/or adjuvant therapy.

## Figures and Tables

**Table 1:** Comparison of the cytotoxicity of the tested compounds in cancer PSN-1 cells and immortalized MS-1 cells *.

Compound	PSN-1 *	MS-1 *
OA	>150	>150
2c	>150	>150
2d	126.56 ± 1.22	>150
IND	>150	>150
3c	87.55 ± 4.87	97.50 ± 1.49
3d	71.89 ± 2.98	93.75 ± 0.85
DCL	>150	>150
4c	103.13 ± 0.28	125.00 ± 2.44
4d	55.21 ± 1.97	69.50 ± 1.83

* IC_50_ values ± SEM (µM).

**Table 2:** Primers used in the R-T PCR.

Gene	Forward Primer	Reverse Primer
*NF-ĸB p50*	5′ATCATCCACCTTCATTCTCAA	5′AATCCTCCACCACATCTTCC
*NF-ĸB p65*	5′CGCCTGTCCTTTCTCATC	5′ACCTCAATGTCCTCTTTCTG
*COX-2*	5′CCTGTGCCTGATGATTGC	5′CAGCCCGTTGGTGAAAGC
*Nrf2*	5′ATTGCTACTAATCAGGCTCAG	5′GTTTGGCTTCTGGACTTGG
*SOD-1*	5′CGACAGAAGGAAAGTAATG	5′TGGATAGAGGATTAAAGTGAGG
*NQO1*	5′CAATTCAGAGTGGCATTC	5′GAAGTTTAGGTCAAAGAGG
*GSTA*	5′GGGAAAGACATAAAGGAGAGAG	5′TCAAAGGCAGGGAAGTAGC
*HO-1*	5′CAGGCAGAGGGTGATAGAAGAG	5′GGAGCGGGTGTTGAGTGG
*GPx*	5′CAACCAGTTTGGGCATCAG	5′TTCACCTCGCACTTCTCG
*PBGD*	5′TCAGATAGCATACAAGAGACC	5′TGGAATGTTACGAGCAGTG
*TBP*	5′GGCACCACTCCACTGTATC	5′GGGATTATATTCGGCGTTTCG

## Data Availability

Data will be available if requested.
